# Differential Gene Expression Reflects Morphological Characteristics and Physiological Processes in Rice Immunity against Blast Pathogen *Magnaporthe oryzae*

**DOI:** 10.1371/journal.pone.0126188

**Published:** 2015-05-22

**Authors:** Parisa Azizi, Mohd Y. Rafii, Maziah Mahmood, Siti N. A. Abdullah, Mohamed M. Hanafi, Naghmeh Nejat, Muhammad A. Latif, Mahbod Sahebi

**Affiliations:** 1 Laboratory of Food Crops, Institute of Tropical Agriculture, Universiti Putra Malaysia, Serdang, Selangor, Malaysia; 2 Department of Crop Science, Faculty of Agriculture, Universiti Putra Malaysia, 43400 UPM, Serdang, Selangor, Malaysia; 3 Department of Biochemistry, Faculty of Biotechnology and Biomolecular Science, Universiti Putra Malaysia, Serdang, Selangor, Malaysia; 4 Laboratory of Plantation Crop, Institute of Tropical Agriculture, Universiti Putra Malaysia, Serdang, Selangor, Malaysia; 5 Plant Pathology Division, Bangladesh Rice Research Institute, Gazipur-1701, Bangladesh; Fujian Agriculture and Forestry University, CHINA

## Abstract

The rice blast fungus *Magnaporthe oryzae* is a serious pathogen that jeopardises the world’s most important food-security crop. Ten common Malaysian rice varieties were examined for their morphological, physiological and genomic responses to this rice blast pathogen. qPCR quantification was used to assess the growth of the pathogen population in resistant and susceptible rice varieties. The chlorophyll content and photosynthesis were also measured to further understand the disruptive effects that *M*. *oryzae* has on infected plants of these varieties. Real-time PCR was used to explore the differential expression of eight blast resistance genes among the ten local varieties. Blast disease has destructive effects on the growth of rice, and the findings of our study provide evidence that the *Pikh*, *Pi9*, *Pi21*, and *Osw45* genes are involved in defence responses in the leaves of Malaysian rice at 31 h after inoculation with *M*. *oryzae* pathotype P7.2. Both the chlorophyll content and photosynthesis were reduced, but the levels of *Pikh* gene expression remained constant in susceptible varieties, with a developed pathogen population and mild or severe symptoms. The *Pi9*, *Pi21*, and *Osw45* genes, however, were simultaneously upregulated in infected rice plants. Therefore, the presence of the *Pikh*, *Pi9*, *Pi21*, and *Osw45* genes in the germplasm is useful for improving the resistance of rice varieties.

## Introduction

Rice (*Oryzasativa* L.) is the second most widely cultivated crop in the world, and due to the growing global population, the production of rice is expected to increase by 40% by 2030. Hence, the production of rice varieties with potential and more stable yield is indispensable for overcoming grain yield reductions and arable land limitations [1]. However, the stability of the rice yield is affected by biotic and abiotic stresses. Rice blast,caused by *Magnaporthe oryzae*, is one of the most calamitous diseases for thiscrop, reducing the annual grain yield by approximately 10–30% [2]. It has injured different parts of plants such as leaf, collar, node, neck and panicle by production of spores and penetration of infection [3]. Blast disease also has destructive effects on the physiological aspects of rice growth; leaf blast disease decreases photosynthesis by reducing the green leaf area and affecting photosynthesis in the regions surrounding lesions [4]. Various control strategies have been applied to overcome rice blast disease, including the use ofresistant varieties [5]. To date, over 85 blast resistance genes and 300 QTLs have been identified, of which 20 have been cloned. Rice blast can be categorised in a gene-for-gene system, in whichone or more similar motifs are encoded by*R* genes[6]. These similar motifs consist of kinase domains, nucleotide binding sites (NBSs), and leucine-rich repeat segments [7]. Among the cloned blast resistance genes, only *Pid2* and *Pi21* encode a receptor that is a kinase [8] and proline-rich protein [9], respectively; the other genes are classified as containing nucleotide binding site-leucinerich repeats (NBS-LRRs) [10]. *Pib*,an NBS-LRR, is a member of a dominant small family gene and confers high resistance to blast fungi in rice plants [11]. The expression of *Pib* is induced at 12 and 24 h after *M*. *Oryzae* inoculation but returns to basal levels at 96 h post-inoculation [12]. In addition, the genes *Pikh* and *Pita* induce broad-spectrum resistance to blast disease [13]. *Pita* is a single-copy gene with low constitutive expression that is the same in both resistant and susceptible rice plants prior to inoculation with *M*. *oryzae* [14]. The *Pi9* and *Os11gRGA8* genes also belong to the NBS-LRR class of proteins and are found in indica rice lines, e.g., 75-1-127, and Peh-kuh-tsao-tu lines, respectively [15], and *Os11gRGA8* is tightly linked to the *Pia* gene on chromosome 11 [16]. The majority of the cloned and mapped *R* genes confer resistance to only specific isolates due to race specificity, though it has been reported that cloning these genes into any rice strain does confer resistance. Nevertheless, identifying *R* genes that have broad-spectrum resistance or their loci will provide essential genetic resources for rice breeding programmes [17–19]. Members of the *WRKY* gene family are transcription factors that bind to the promoter of W-box defence genes to regulate their transcription [20,21], and many *WRKY* genes are expressed in response to pathogen infections, such as blast fungi and bacterial blight diseases [22–24]. Indeed, an over-expression analysis of *WRKY45* and *WRKY22* genes revealed the induction of a resistant phenotype following blast inoculation [25]. Although many resistant varieties are available from IRRI’s vast collection of rice germplasm, some rice varieties that were released as resistant became susceptible after only a few years of cultivation due to blast fungal evolution and adaptation [26]. Therefore, screening rice varieties based on their resistance to blast disease and identifying the most important genes involved in the defence response are essential for improving rice crops. To better investigate plant-fungal interactions, a rapid and precise measurement of fungal growth in plants is needed to properly assess both fungal pathogenicity and host resistance levels. Several methods have been used to identify and quantify fungal pathogens in the environment (plants and soil). For instance, the overall fungal biomass in the environment has been approximated by calculating the amount of fungal compounds such as ergosterol or even chitin [27–29]. However, these methods often lack specificity and usually do not correlate with the actual fungal biomass due to fungal sporulation and disturbances of enzymatic reactions in plants [30]. In addition, numerous studies have used quantitative PCR to calculate the amount of plant pathogenic fungus during plant-fungal interactions [31,32], yet this type of quantitative information is not produced directly from target DNA but instead is derived from the similarity rate between target and non-target DNA.

In this study, the expression profiles of 8 selected genes involved in defence responses to blast disease among 10 common Malaysian rice varieties were for the first time examined using real-time PCR (RT-qPCR). Two gene groups, *Pikh*[33], *Pib*[12], *Pi21* [9], *Pia* (*Os11gRGA8*) [16], *Pita* [34,35] and *Pi9* [36] (major genes confer high resistance to blast fungal), and also *OsWRKY22* [37] and *OsWRKY45* [24] (involving in mitogen-associated protein kinase (MAPK) [38] and salicylic acid (SA) signalling [24,39], cascades and transcriptional re-programming in plant/pathogen interactions) were selected to be investigated. MAPK and SA signalling cascades are the earliest signalling events after plant sensing of the invading pathogen as they link the perception of external stress to cellular responses [38,40]. Furthermore, we investigated correlations among gene expression levels, leaf blast severity, and photosynthesis and its components (chlorophyll a and b) in the visible lesion area of inoculated plants. The fungal population was also quantified and compared between susceptible and resistant plants using a qPCR quantification technique.

## Experimental Procedure

### Plant material and fungal isolate

Ten important Malaysian rice varieties and an *M*. *oryzae* strain (P7.2) were provided by the Malaysian Agricultural Research and Development Institute (MARDI) (Table 1). Rice seeds were soaked in water for three days and germinated on moist Whatman filter paper in Petri dishes. Subsequently, the seeds were planted in pots using autoclaved potting soil, transferred to a glasshouse and kept at 25–30°C for three weeks.

**Table 1:** Selected characteristics of ten Malaysian rice varieties.

Variety	Yield (t/ha)	Leaf blight disease(*Xanthomonasoryzea*)	Brown planthopper	Sheath blight (*Thanatephoruscucumeris*)
MR159	3–5.4	Resistant	Semi-resistant	Susceptible
PH9	3.8–4.7	Susceptible	Semi-susceptible	Resistant
MR84	4–6.2	Susceptible	Semi-susceptible	-
MR185	6–9.2	Resistant	Semi- resistant	-
MR232	6.5–8.7	Semi-resistant	Semi-susceptible	Susceptible
MR253	6–7.5	Semi-resistant	Semi-resistant	Semi-resistant
MR219	6.5–10.7	Resistant	-	-
MR263	6–7.5	Semi-resistant	Semi-resistant	Semi-resistant
MR269	6–7.5	Semi-resistant	Semi-resistant	Semi-resistant
MRQ74	4.5–5.5	Semi-resistant	Semi-resistant	-

### Plant inoculation and disease severity assay

**Fig 1:**
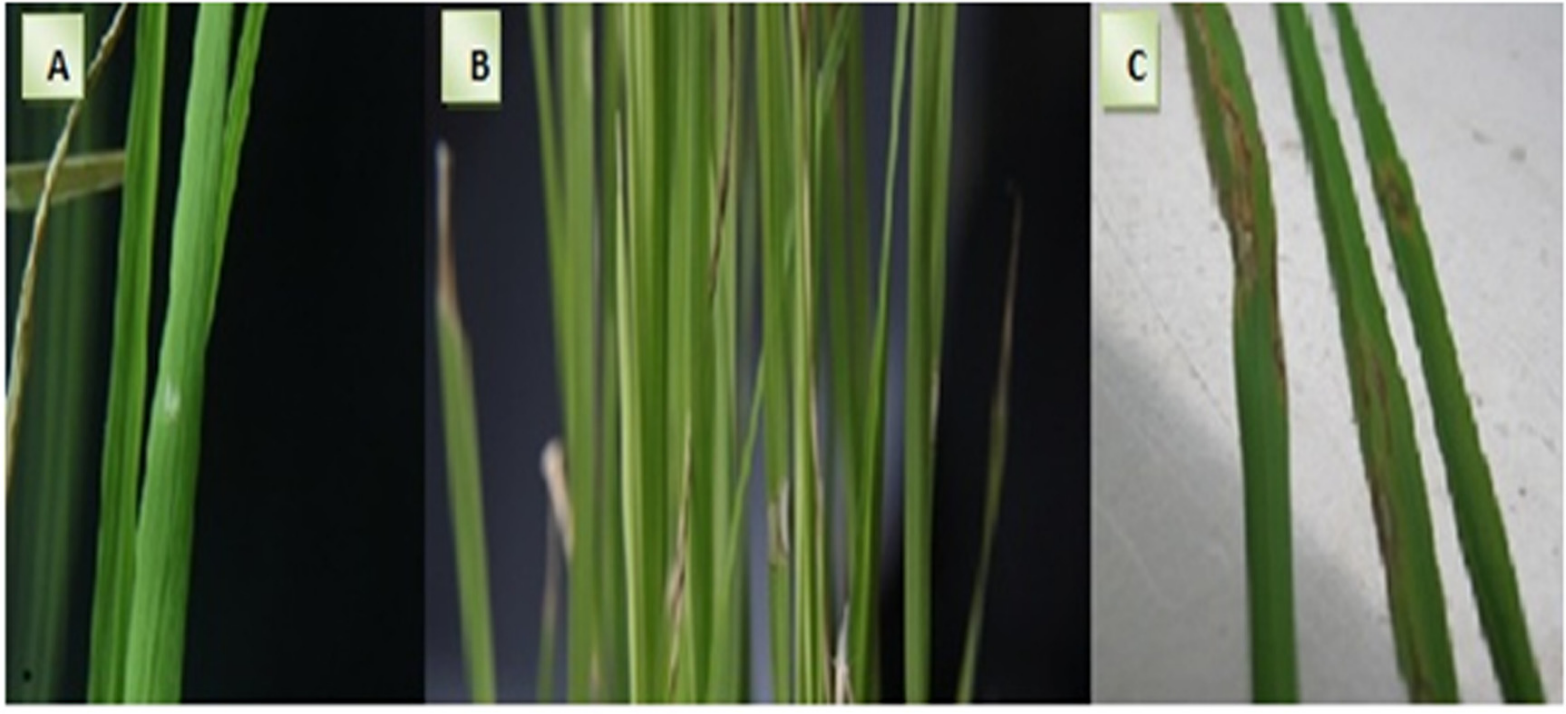
Blast disease symptoms caused by *M*. *oryzae* on the leaves of susceptible rice varieties. A. Symptoms at 3 days after inoculation. B and C. Disease severity in rice seedling at 7 days after inoculation.

Three-week-old rice seedlings at the three-leaf stage were inoculated with an *M*. *oryzae* spore suspension containing 3×10^5^ spores/mL.The inoculation experiments were repeated three times for all rice varieties. We measured three components of partial resistance: the blast lesion degree (BLD), blast lesion type (BLT), and percent disease leaf area (%DLA). Two traits, %DLA and BLT, were estimated and scored based on the Correa-Victoria and Zeigler methods [41]. Disease severity (Fig 1) was recorded at 7 days after inoculation (DAI) using a 0 to 9 disease assessment scale: 0, no signs of infection; 1, brown specks ˂ 0.5 mm in diameter; 3, brown spots 0.5–1 mm wide; 5, round to ovate-shaped lesions and 1–3 mm in diameter with a grey centre, 7: spindle-shaped lesions with a grey centre; and 9, combinations of small, white, grey, or bluish lesions without distinct borders. The %DLA was scored as follows: 0, highly resistant and no symptoms; 1–2, lesions 1–2 mm and no sporulation; 3, lesions 2–3 mm size and little sporulation; and 4, spindle-shaped lesions more than 3 mm in size with heavy sporulation. The BLD scoring was implemented based on the Standard Evaluation System (SES) of the International Rice Research Institute [42].

### Differentiation of pathogen population between inoculated susceptible and resistant plants using a bacterial quantification technique

#### Real-time PCR assay

For the real-time PCR assay, total DNA was extracted from three-week-old susceptible (MR232, MR219 and MR263) and resistant seedlings, which hadbeen inoculated with *M*. *oryzae* pathotype P7.2 at a concentration of 3×10^5^ spores/mL, at 31, 48 and 72 h post-infection using the i-genomic BYF DNA extraction mini kit (EBN, Route d'Esneux, Dolembreux, Belgium). The absolute quantification of the *M*. *oryzae* population in inoculated rice plants was carried out based on the real-time PCR standard curve method. The standard curves were constructed using the copy number of the *28S rDNA* gene plotted against the quantification cycle (Cq) obtained from 10-fold serial dilutions of the PCR products from a pure fungal culture. All preparing proceduresof standard curve, ranging from DNA extraction to the purification of specific bands (*28S rDNA* gene), are described below.

#### DNA extraction from fungal cultures

About 0.2 g of fungal mycelia previously cultured in potato dextrose broth was ground with liquid nitrogen and fungal DNA was extracted using modified CTAB method [43].

#### Primer design and *28SrDNA* isolation from the fungal genome

A specific pair of DNA primers was designed based on the 3' end of the *M*. *oryzae 28SrDNA* gene (Table 2). More than 100 copies of the *28SrDNA* gene are present in the *M*. *oryzae* genome and are constitutively and highly expressed [44,45]. The PCR programme was performed using a Taq DNA polymerase kit (Fermentas, Waltham, Massachusetts, USA) with the following parameters: initial denaturation at 95°C for 4 min, 40 cycles of 95°C for 30 s, 60°C for 30 s, and 72°C for 1 min, and a final extension at 72°C for 5 min. The PCR product was isolated on a 1% agarose gel and stained with ethidium bromide. Finally, the expected *28SrDNA* gene band (approximately 330 bp) was purified using a QIAprepspin miniprep kit (Qiagen, Hilden, North Rhine-Westphalia, Germany).

**Table 2:** Primers for targeting*M*. *oryzae* used for real-time PCR.

Target group	Sequence 5^/^–3^/^	
***28S rDNA***	Forward	TACGAGAGGAACCGCTCATTCAGATAATTA
	Reverse	TCAGCAGATCGTAACGATAAAGCTACTC

The purity and concentration of the *28S rDNA* gene in each sample was measured using a Nanodrop ND-1000 spectrophotometer (ImplenNanoPhotometer, Munich, Bavaria, Germany). The number of copies of *28S rDNA* per mL of elution buffer was calculated using the following formula (available online in the Genomics and Sequencing Centre web-based calculator of the University of Rhode Island) [46].

$$\text{Number of copies}=\frac{\text{Amount of DNA}\;\left(\frac{\mu \text{g}}{\text{mL}}\right)\times 6.022\;\times 10^{23}}{\text{Length}\;\left(\text{bp}\right)\times \;10^{9}\;\times 650}$$

Because the efficiency of amplification among primers and templates can vary, the amplification efficiency (E) of each primer-template combination was determined based on the slope value of the linear regression of each standard curve calculated by the following equation:

$$\text{E}\left(\%\right)=\left[10^{\left(-\frac{1}{\text{Slope}}\right)}-1\right]\times 100$$

Real-time PCR was performed using aBioRad CFX96 Real-time PCR system(BioRad, Hercules, California, USA)with optical-grade plates. The primers used in the quantification of different fungal populations are shown in Table 2. The real-time PCR reaction was performed in a total volume of 25 μL using the QiagenQuantifast SYBR Green PCR, USA. Each sample was amplified in triplicate. A no-template control (NTC) was included to rule out any cross-contamination. The real-time PCR cycling conditions consisted of 5 min at 95°C, followed by 40 cycles of 10 s at 95°C and 30 s at 60°C. Upon the completion of amplification, the specificity of the product was confirmed by melting curve analysis. The real-time PCR products were incubated by raising the temperature from 70 to 95°C in 0.5°C increments, with a hold of 5 s at each increment.

### Chlorophyll content

The leaves of treated and untreated plants were collected at seven days after inoculation. The chlorophyll content was estimated using the method of Witham et al. [47]. The absorbance of the solution was recorded at 645 and 663 nm using a scanning spectrophotometer (AL800, Aqualytic, Germany). The chlorophyll content was measured as mg/g^-1^ of the sample using the following formulae:

$$\text{Chlorophyll a}\;\left(\frac{\text{mg}}{\text{g}}\text{fresh leaf}\right)=12.7\;\left(\text{A}663\right)-\;2.69\frac{\left(\text{A}645\right)}{1000}\times \text{V}/\text{W}$$

$$\text{Chlorophyll b}\;\left(\frac{\text{mg}}{\text{g}}\text{fresh leaf}\right)=22.9\;\left(\text{A}645\right)-\;4.68\frac{\left(\text{A}663\right)}{1000}\times \text{V}/\text{W}$$

$$\text{Total chlorophyll}\;\left(\frac{\text{mg}}{\text{g}}\text{fresh leaf}\right)=20.2\;\left(\text{A}645\right)+\;8.02\frac{\left(\text{A}663\right)}{1000}\times \text{V}/\text{W}$$

where A645 and A663 are the absorbance of the solution at 645 and 663 nm, respectively, V is the volume of the solution in mL, W is the weight of the fresh leaf sample in mg, and 12.7, 2.69, 22.9, 4.86, 20.2 and 8.02 are absorption coefficients.

### Photosynthesis analysis

Seven days after inoculation, the amount of photosynthesis was measured among treated and control varieties using the LI-6400XT Portable photosynthesis system (LI-COR, Lincoln, Nebraska, USA).

### Real time-PCR analysis

Specific primers were designed for the rice resistance genes available in the GenBank database using the primer premier6 software (Table 3). The *18S rRNA* and *tubulin* reference genes of *O*. *sativa* were used as expression control genes for normalisation.

**Table 3:** List of primers for 10 genes used for Real time-PCR.

List of genes	Forward primer (5´- 3´)	Reverse primer (5´- 3´)
*Pikh*	AAGATTTTCGAGGCTCTTCTCTA	ATGAATCTGTTTCCTCGTCTTG
*Pib*	GGGAAAAATGGAAATGTGC	AGTAACCTTCTGCTGCCCAA
*Pita*	ATTATTGAGCTTCTTTCTTTCTCTG	GAAACAAAATTACCTCTACTCTGAAG
*Pi21*	GGTCATCTTGGTGGACCTGCAATG	CGATGCAGTACTCCTCTTCAAGGC
*Pi9*	TGCCCAACCTTTACCCACTGTA	AACATGAGTAGAAACAAATTAGTTTG
*OSWRKY22*	CGGCGGAAAGACTGAGAAAG	CAGCCTCTGATGACGGTGAG
*OSWRKY45*	ACGACGAGGTTGTCTTCGATCTG	GCCCGTGTCCATCCATGATTCTTC
*Os11gRGA8*	CAAGCCAAGTTCCAC	TGCACTCTGCATGTTGTTCA
*18SrRNA*	ATGATAACTCGACGGATCGC	CTTGGATGTGGTAGCCGTTT
*α-Tubulin*	GGAAATACATGGCTTGCTGCTT	TCTCTTCGTCTTGATGGTTGCA

At 31 h after inoculation, a time when the blast fungus emerged through the cell wall and entered into the epidermal cell [48], RNA was extracted from the leaves of infected and control plants using TRIzol Reagent (Invitrogen, USA)[49]. The expression levels of eight blastresistance genes (*Pikh*, *Pib*, *Pita*, *Pi21*,*Pi9*,*Os11gRGA8*, *OsWRKY22* and *OsWRKY45*)were evaluated by real-time PCR.

A 1-μL aliquot of RNA from each sample was used to prepare 20-μL reactions (based on the KAPA SYBER FAST One-Step RT-qPCR, Wilmington, Delaware, USA). The cycling conditions were a primary incubation of 5 min at 42°C and 5 min at 95°C, followed by 40 cycles of 95°C for 3 sec, 60°C for 30 sec, and 72°C for 3 sec. In this part of the study, all data were derived from three independent biological replicates. Amplicon specificity was checked using a melting curve analysis after 40 cycles by increasing the temperature from 60°C to 95°C.

### Data analysis

The collected data were standardised and statistically analysed using the R software version 3.0.2 (R Core Team,Vienna, Austria). The comparison ofmeans was performed using the Duncan test. Cluster analysis based on similarity matrices was employed for the morphological and physiological data. The Relative Expression Software Tool(REST) (Qiagen, Hilden, North Rhine-Westphalia, Germany) was used to analyse the real-time amplification data.

## Results

### Severity assessment

Seven of the 10 rice varieties showed a high degree of resistance to pathotype P7.2 of rice leaf blast in glasshouse tests (Table 4).

**Table 4:** The ANOVA of seven physiological and morphological traits in ten rice varieties.

Source Of Variation	DF	BLT	BLD	DLA	Dif Chlo A	Dif Chlo B	Dif T Chlo	Photo
Block	2	0.000^ns^	0.000^ns^	0.000^ns^	0.169**	0.091^ns^	0.043^ns^	0.004^ns^
Treat	9	9.633**	34.300**	23.741**	5.368**	5.770**	5.561**	1.706**
Error	18	0.000	0.000	0.000	0.0148	0.062	0.012	0.008
Total	29	-	-	-	-	-	-	-
CV	-	1.81E-6	1.48E-06	0	14.111	24.615	12.124	14.947

^ns^ and ** indicate not significant and a significance level of 1%, respectively. Dif Chlo A: difference in chlorophyll A between control and treated plants. Dif Chlo B: difference in chlorophyll B between control and treated plants. Dif T Chlo: difference in total chlorophyll content between control and treated plants. Photo: difference in photosynthesis between control and treated plants. BLT: Blast lesion type. BLD: Blast lesion degree. DLA: Disease leaf area (%).

**Fig 2:**
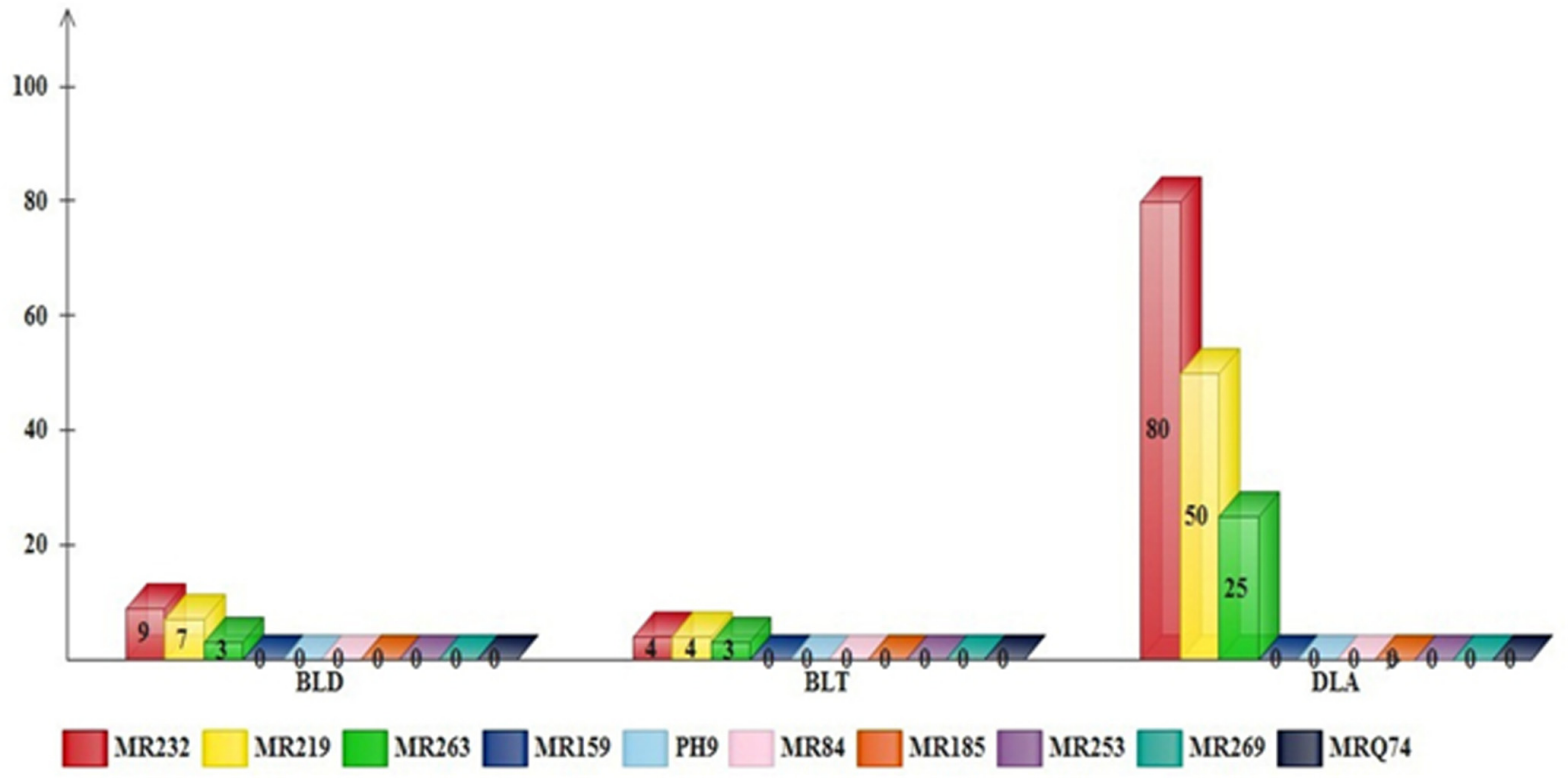
Differences in morphological traits (blast lesions) of rice varieties. BLD: Blast lesion degree. BLT: Blast lesion type. DLA (%): Disease leaf area.

However, three rice varieties exhibited different levels of susceptibility. The results of a comparison of meansusing the Duncan test showed that the highest blast lesion degree (9), blast lesion type (4), and disease leaf area (80%) were found in MR232. Furthermore, MR219 and MR263 scored 7 and 3 for the blast lesion degree and 4 and 3 for the blast lesion type, respectively (Table 5). The percentage of disease leaf area was 50% and 25% in MR219 and MR263, respectively (Fig 2).

**Table 5:** Mean comparison of different morphological and physiological traits in rice varieties.

Varieties	BLT	BLD	DLA	DIFF A	DIFF B	DIFF T	PHOTO
MR159	0 c	0 d	0 d	0.000 e	0.146 c	0.465 d	0.239 d
PH9	0 c	0 d	0 d	0.089 ed	0.298 c	0.187 e	0.158 d
MR84	0 c	0 d	0 d	0.329 d	0.324 c	0.144 e	0.239 d
MR185	0 c	0 d	0 d	0.137 ed	0.241 c	0.000 e	0.260 d
MR232	4 a	9 a	80 a	1.696 c	3.4603 a	4.288 a	2.534 a
MR253	0 c	0 d	0 d	0.097 ed	0.148 c	0.126 e	0.346 d
MR219	4 a	7 b	50 b	2.324b	3.439 a	1.564 c	1.177 b
MR263	3 b	3 c	25 c	3.888a	1.854 b	1.985 b	0.833 c
MR269	0 c	0 d	0 d	0.000 e	0.000 c	0.521 d	0.184 d
MRQ74	0 c	0 d	0 d	0.063 ed	0.226 c	0.020 e	0.170 d
Mean	1.1	1.9	15.5	0.8623	1.01363	0.93	0.614
CV(%)	1.81E-6	1.48E-06	0	14.111	24.615	12.124	14.947

Same letter(s) in a column denote no significant difference at *P ≤ 0*.*05*. DifChlo A: difference in chlorophyll A between control and treated plants. DifChlo B: difference in chlorophyll B between control and treated plants. Dif T Chlo: difference in total chlorophyll content between control and treated plants. Photo: difference in photosynthesis between control and treated plants. BLT: Blast lesion type. BLD: Blast lesion degree. DLA: Disease leaf area (%).

### Real time-PCR quantification between inoculated susceptible and resistant plants

A significant variation was detected in the abundance of*M*. *oryzae* among the inoculated susceptible and resistant plants, including all sample collected at 31, 48 and 72 hours after inoculation. The expected size of PCR amplicon was 330 bp forthe blast fungus*28S rDNA* (S1 Fig and Table 6). The standard curve of the target gene is shown in S2 Fig and was constructed using the plot of copy numbers of *28S rDNA* gene of *M*. *oryzae* against its Cq value. The standard curves had a high correlation coefficient of R^2^ = 0.996, indicating that the Cq value was proportional to the copy numbers of *28S rDNA* gene, for blast fungal. From the slope of the liner regression of -3.353 amplification efficiency obtained was 98.7% for *M*. *oryzae*. A good reaction should have an amplification efficiency of between 90–110% and R^2^ value should be ≥ 0.985,and in this study, the amplification efficiencies of 98.7%, and R^2^ values of 0.996 for the standard curves of *M*. *oryzae* was within the acceptable range.

**Table 6:** Concentration and purity of extracted*M*. *oryzae* DNA.

Sample	Concentration (ng/μL)	Purity	
		260/280	260/230
Extracted fungal DNA	3700.2	2.18	1.12
DNA extracted from the gel	94	-	-

The amplification curve for *M*. *oryzae* (S3 Fig) was constructed by plotting the cycle numbers against the fluorescence signal (RFUs, relative fluorescence units). No fluorescence signals were detected from the no-template control.

The melting curve for the blast fungal is shown in S4 Fig. From the result, the melting temperature of 86.5 was detected at which the set of primer was specific for estimation of *M*. *oryzae*.

**Fig 3:**
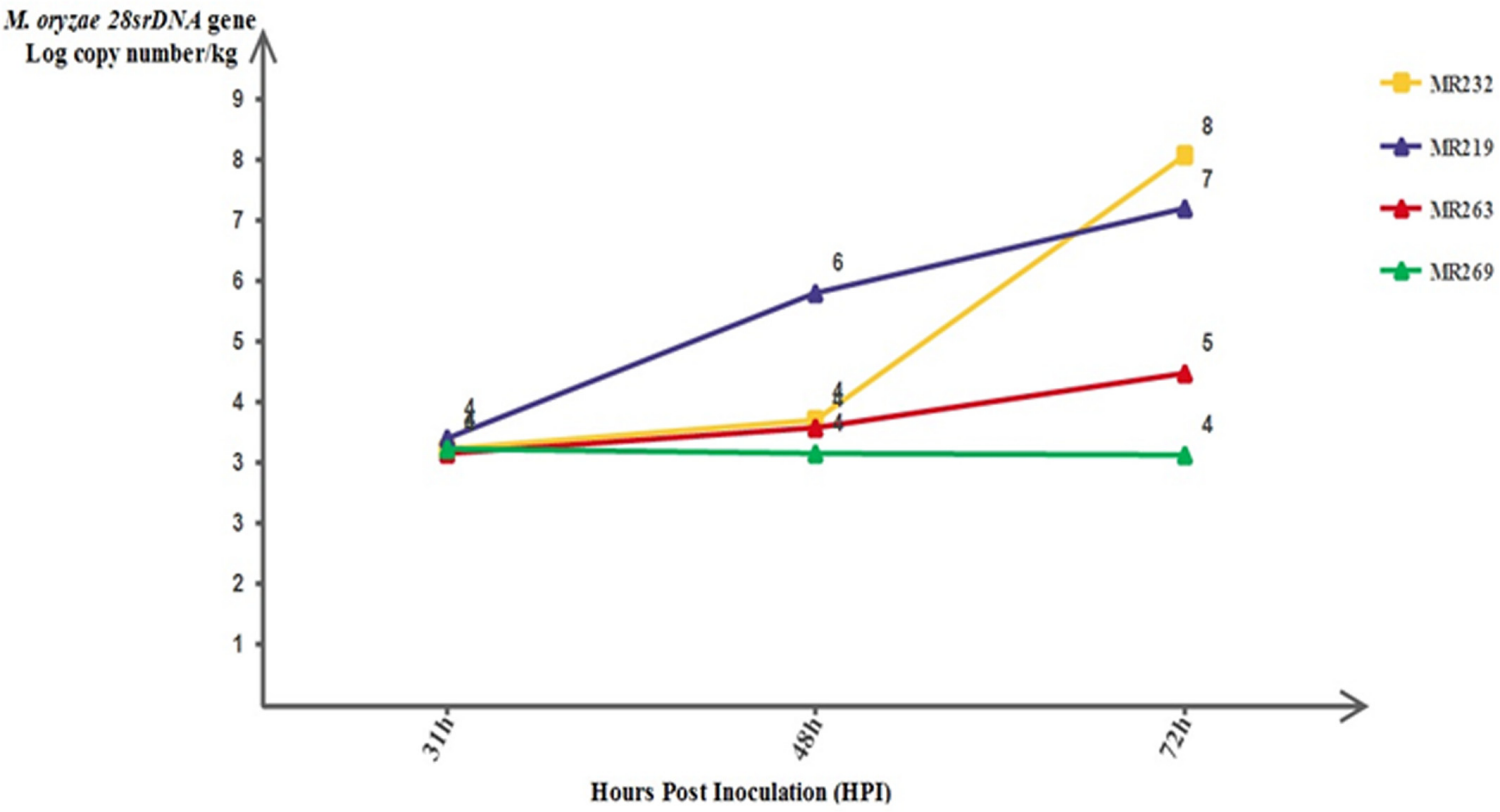
A comparison of *M*. *oryzae* populations between resistant and susceptible plants at 31, 48 and 72 hours after infection, as quantified using real-time PCR. Bars represent the means of three biological samples at each time point.

The real-time PCR quantification results for the *M*. *oryzae* populationsfrom inoculated susceptible and resistant plants at 31, 48 and 72 hours after inoculation are shown in Fig 3. The blast fungal population were significantly (*P < 0*.*05*) higher in susceptible plants compared with those observed in resistant plants at 48 and 72 hours after inoculation.

The *M*. *oryzae* populations quantified by real-time PCR are shown in Fig 3. At 48 and 72 hours after inoculation, the fungal populations in the susceptible plants were significantly (*P*< 0.05) higher (4.116 and 7.914 in MR232; 5.944 and 7.151 in MR219; 4 and 4.781 in MR263, respectively, for the log *28S rDNA* gene copy number/kg) than their populations in resistant plants (3.628 and 3.609,respectively, for thelog*28S rDNA* gene copy number/kg). However, at 31h of inoculation, the blast fungal population in the resistant plants (3.510 log *28S rDNA* gene copy number/kg) was not significantly different from that of the susceptible plants (3.706, 3.848 and 3.628 log *28S rDNA* gene copy number/kg for MR232, MR219 and MR263, respectively).

### Chlorophyll content

**Fig 4:**
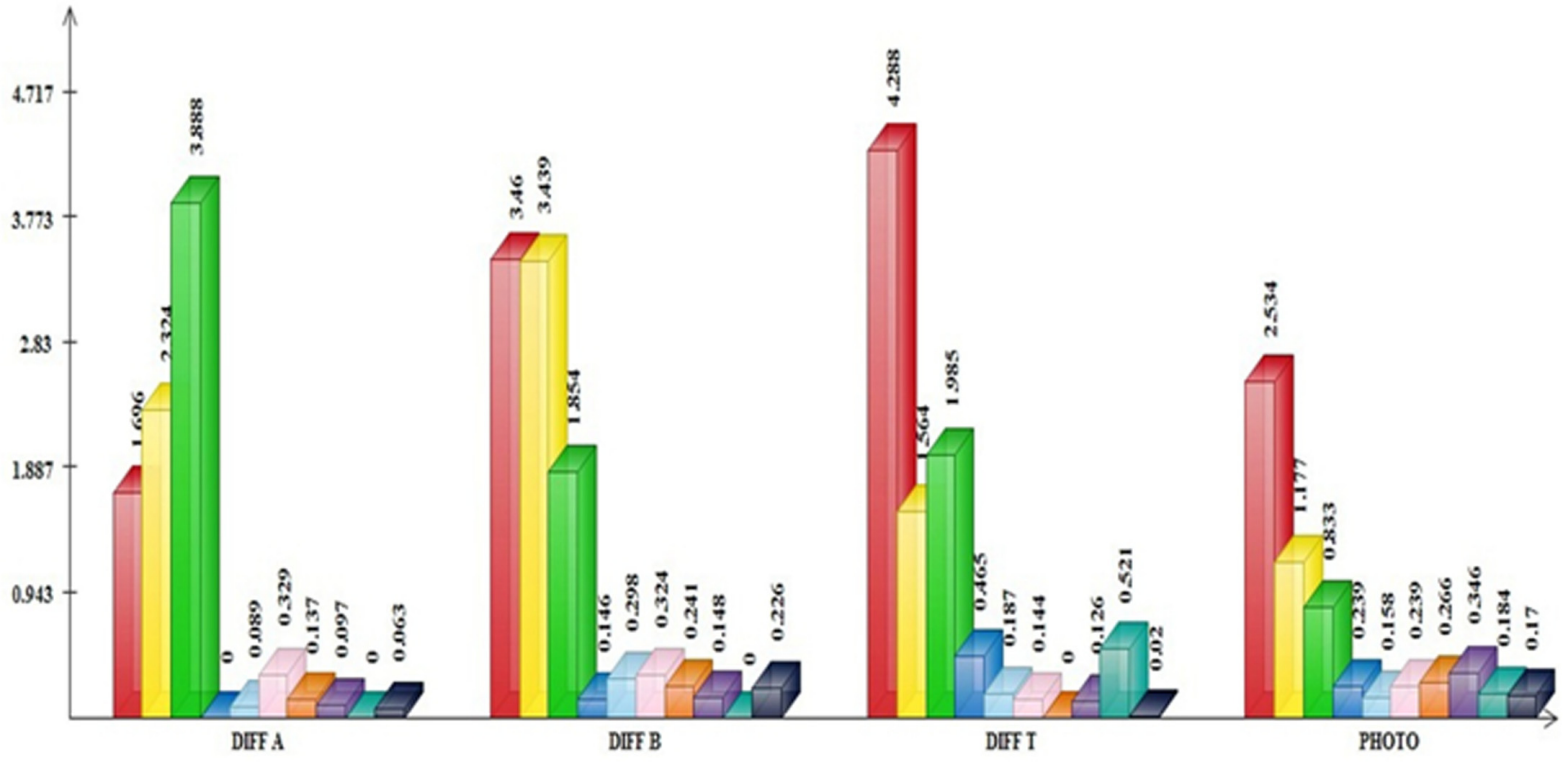
Variations in physiological traits among ten rice varieties. DIFF A: difference in chlorophyll a. DIFF B: difference in chlorophyll b. DIFF T: difference in total chlorophyll. PHOTO: difference in photosynthesis between control and treated plants.

The chlorophyll contents (total, a and b) showed significant differences in 5% level of probability among ten varieties (Table 4). The highest decrease rate of chlorophyll a content was obtained in infected MR263 compared with the control. However, the maximum reduction rate of chlorophyll b and total chlorophyll contents in infected plants wasfound for MR232 (Table 5 and Fig 4).

### Photosynthesis analysis

The effects of the blast pathogen in reducing leaves photosynthesis in inoculated plants were significant among the rice varieties (Table 4). The most decreased level of photosynthesis was revealed in MR232. Besides, in comparison to control plants, the diminished photosynthesis ratio was observed in inoculated MR219 and MR263, respectively (Table 5 and Fig 4). On the other hand, the variationsin photosynthesis between the treated and control plants were not significant in the remainder varieties.

### Correlation analysis

Determination of the correlations between different traits, especially photosynthesis and chlorophyll components, allow for the identification of interactions amongaffected traits in rice plants infected with *M*. *oryzae*. Blast lesion type, disease leaf area and the blast lesion degree showed a negative correlation with photosynthesis and chlorophyll components. The highest correlation coefficient was observed between the blast lesion type and disease leaf area (r = 0.962). Chlorophyll b, in comparison to chlorophyll a,represented a larger proportion of the total chlorophyll content and photosynthesis (r = 0.929 and 0.801, respectively) ininfected rice leaves. The changes in blast lesion type and disease leaf area were able negatively to manage the amount of photosynthesis (r = -0.916 and -0.891) (S1 Table).

### Cluster analysis

**Fig 5:**
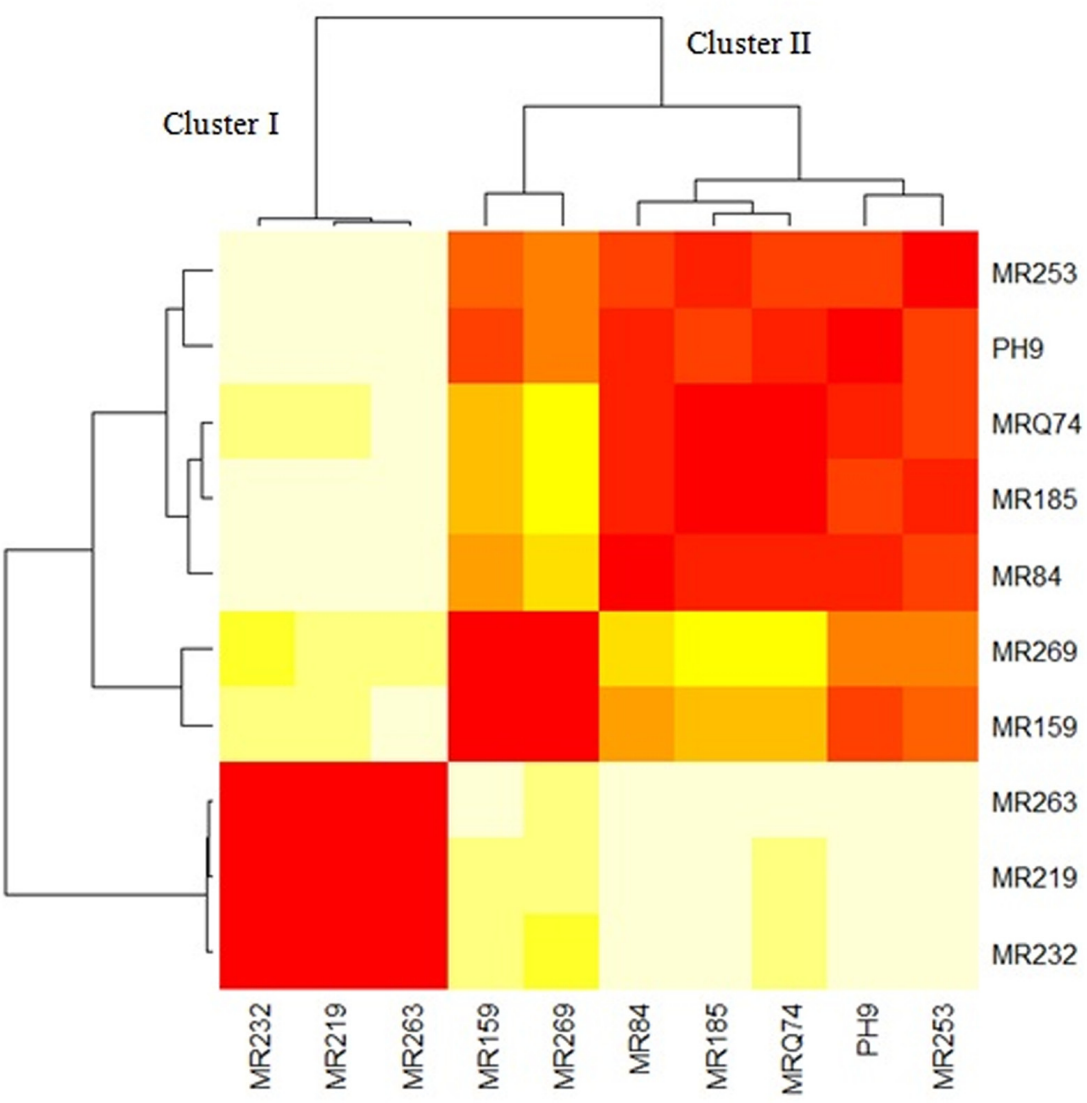
Cluster of ten rice varieties based on morphological and physiological traits.

The ten Malaysian rice varieties were categorized to two clusters based on seven morphological and physiological characters (Fig 5). Cluster I is composed of the susceptible rice varieties MR232, MR263 and MR219 to blast disease. While, cluster II is collected of the resistant varieties.

### Gene expression study

Gene expression studyof eight blast resistance genes was performed simultaneously in an attempt to identify statistically up-regulated genes in the most common and popular rice varieties infected by *M*. *oryzae* in Malaysia.

**Fig 6:**
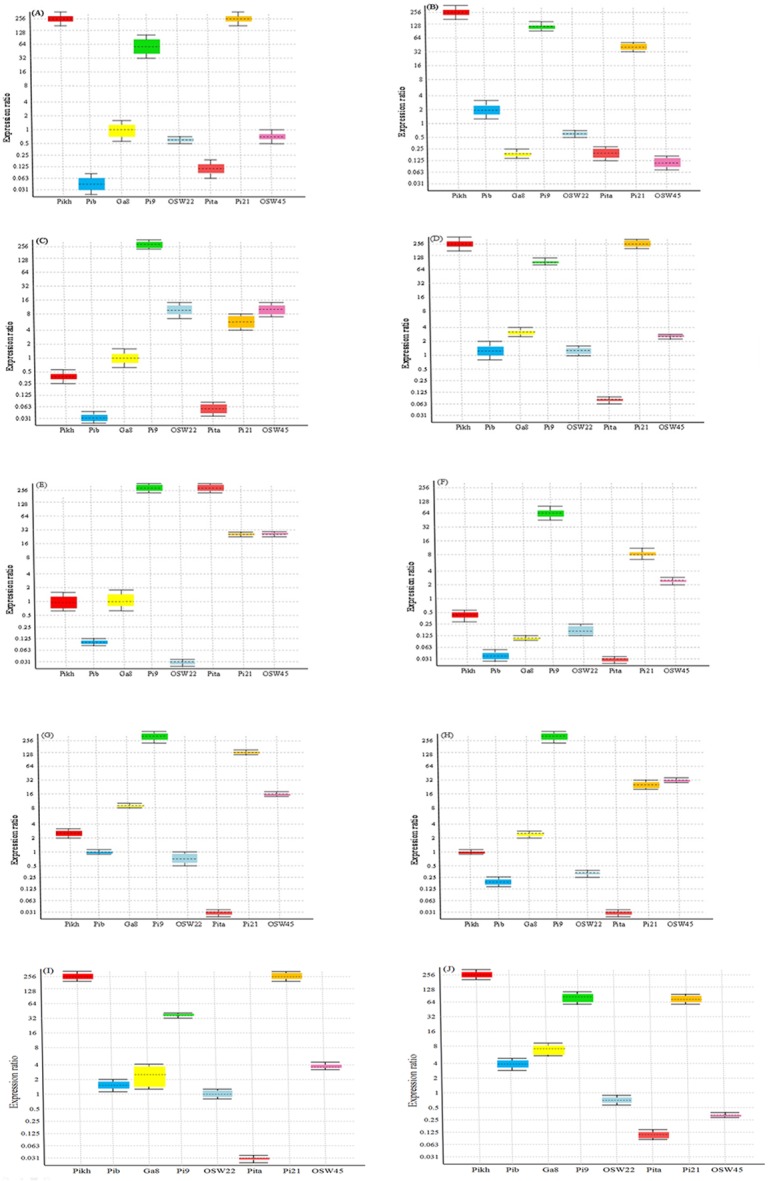
Relative expression levels of *Pikh*, *Pib*,*Os11gRGA8(GA8)*, *OsWRKY22 (Osw22)*, *Pita*, *Pi21* and *OsWRKY45 (Osw45)* genes calibrated using *18SrRNA* and *tubulin* as reference genes in infected and control rice plants using relative quantitative real-time PCR. Expression levels of 8 genes in 10 infected varieties (A) MR159, (B) PH9, (C) MR84, (D) MR185, (E) MR232, (F) MR253, (G) MR219, (H) MR263, (I) MRQ74, and (J) MR269.

RT-qPCR was used to explore the variabilityin gene expression between control and *M*. *oryzae-*infected rice plants. Various expression patterns of *Pikh*, *Pib*, *GA8*, *OsW22* and *OsW45* genes have been found at 31 h after inoculation of rice varieties (Table 7). *Pikh* gene was up-regulated significantly in MR159, PH9, MR185, MR269 and MRQ74, and down-regulated in MR84 and MR253, while relative expression of this gene was constant in MR232, MR219 and MR263 varieties. On the other hand, *Pita* gene showed significantly lower expression in ten infected varieties compared with control. Whereas, the transcript level of *Pi9* and *Pi21* genes significantly increased in the leaves of all varieties after 31 hours of inoculation (Fig 6). Furthermore, the expression level of *OsW45* was higher in the MR185, MR232, MR219, MR263, and MRQ74 varieties at 31 h post-inoculation, whereas the expression of *GA8*, *Pib* and *OsW22* was down-regulatedin most of the rice varieties.

**Table 7:** Relative expression patterns and *P*-values of 8 genes in 10 rice varieties.

Genes	MR159	PH9	MR84	MR185	MR232	MR253	MR219	MR263	MR269	MRQ74
*PikhP*-value	UP	UP	DOWN	UP	-	DOWN	-	-	UP	UP
	0.000	0.000	0.000	0.000	0.650	0.000	0.161	0.822	0.000	0.000
*PibP*-value	DOWN	-	DOWN	-	DOWN	DOWN	-	DOWN	UP	UP
	0.000	0.170	0.000	0.499	0.000	0.000	0.501	0.000	0.000	0.000
*GA8P*-value	-	-	-	UP	-	DOWN	UP	-	-	-
	0.661	0.169	0.834	0.000	0.650	0.000	0.000	0.162	0.154	0.837
*Pi9P*-value	UP	UP	UP	UP	UP	UP	UP	UP	UP	UP
	0.000	0.000	0.000	0.000	0.000	0.000	0.000	0.000	0.000	0.000
*Osw22P*-value	-	-	UP	-	DOWN	DOWN	-	DOWN	DOWN	-
	0.169	0.169	0.000	0.827	0.000	0.000	0.502	0.000	0.000	0.667
*PitaP*-value	DOWN	-	DOWN	DOWN	DOWN	DOWN	DOWN	-	DOWN	DOWN
	0.000	0.169	0.000	0.000	0.000	0.000	0.000	0.162	0.000	0.000
*Pi21P*-value	UP	UP	UP	UP	UP	UP	UP	UP	UP	UP
	0.000	0.000	0.000	0.000	0.000	0.000	0.000	0.000	0.000	0.000
*Osw45P*-value	-	-	-	UP	UP	-	UP	UP	DOWN	UP
	0.339	0.169	0.164	0.000	0.000	0.177	0.000	0.000	0.000	0.000

UP: Up-regulated gene; DOWN: Down-regulated gene; Dash sings: gene expression was unchanged.

## Discussion

The results of our severity assessment revealed different levels of resistance among the rice varieties. This evaluation confirmed the susceptibility of the MR232, MR219 and MR263 varieties to the *M*. *oryzae* pathotype P7.2. However, it should be noted that the morphological screening of rice plants provides only preliminary results that must be confirmed by further testing. Compared with the common disease scoring that is based on the number and size of blast lesions, the DNA-based real-time PCR technique enables a more precise computation of the relative growth and absolute biomass of *M*. *oryzae*. Additionally, the quantity of fungal growth in the plants may not be continuously proportional to the distribution of disease symptoms elicited by various blast fungal isolates. Thus, both the quantification of fungal growth and the morphological evaluation of disease symptoms are required to more precisely assess the aggressiveness of various blast fungal isolates. In our study, the quantification of fungal populations in inoculated susceptible and resistant plants was confirmed by a morphological analysis. The growth of the fungus and the susceptibility of MR232, MR219 and MR263 were verified, but the fungal growth was constant in the resistant varieties at 31, 48 and 72 h after inoculation. Larger*M*. *oryzae* populations (46 to 80 times) were found in seedling leaves of susceptible cultivars compared with resistant cultivars, as has been previously reported at 4 and 6 days post-inoculation using real-time PCR quantification [41]. In fact, real-time PCR is an excellent tool for *M*. *oryzae* quantification in plants and can be applied for the reliable evaluation of fungal pathogenicity [32].

We found a negative correlation between photosynthesis and its components and the degree of blast lesions (BLD, BLT and DLA) in infected rice plants. Photosynthesis produces sustained energy for a plant and must therefore be incorporated into photogenedefence mechanisms. Indeed, a decrease in chlorophyll contents and photosynthesis in response to infection is a plant defence strategy that is usedin pathogen evasion[50]. The activation of plant defences leads to a rapid decrease in non-photochemical quenching (NPQ) and thus limits the carbon availability tothe pathogen [51]. There are numerous reasons why plant contacts with pathogens can bedetrimental to photosynthesis [52]. The initiation of defence responses requires energy and photo-assimilate as carbon sources to produce defence compounds;consequently, the photosynthesis demands in the plant increase [53]. Furthermore, sugars, as carbon compounds, are consumed by pathogens. Despite the predicted increase, the infection of plants by pathogens often leads to a reduction in photosynthesis [54–58].

Quantitative real-time PCR (RT-qPCR) is a multipurpose technique for the precise, sensitive, reliable and high-throughput detection and quantification of a target sequence in various samples [59].

In this study, we performed RT-qPCR for eight defence response genes to identify resistance genes with increased expression levels in 10 commonly grown local rice varieties. Gene expression study through simultaneous measurement of the activity of several genes can elucidate how cells react to a particular treatment. The simultaneously increasing transcript levels of *Pikh*, *Pi9*, *Pi21*, and *Osw45* among most of the rice varieties highlight their importance in immunity against *M*. *oryzae* at31 h after inoculation. However, the over-expression of *Pi9*, *Pi21* and *Osw45* is valuable when it is accompanied by the upregulation of *Pikh* expression. We hypothesise that the absence of resistance to blast fungus pathotype P7.2 in susceptible rice varieties is due to a lack of *Pikh* transcript upregulation. When the blast fungus enters the plant, the plant’s immune system is activated, and energy sources (carbon) are compromised. This process promotes catabolic pathways and reduces cell growth and proliferation by switching off protein, carbohydrate and lipid biosyntheses [60]. The activation of these genes at the same time could prevent this deficiency in resistant varieties by producing proteins that are involved in the innate immune system. The recovered immune system does not need to consume more carbon as an energy source. Therefore, and as our experiments show, the decrease in photosynthesis and chlorophyll content in resistant rice plants is not significant. However, compared with control plants, the amount of chlorophyll and photosynthesis are reduced in susceptible rice plants. The over-expression of *Pikh*, *Pi9* and *OsWRKY45* genes in transgenic rice plants after inoculation confers extremely strong resistance against blast disease [25,33,36]. Moreover, the over-expression of the *Pi21* transcript successfully induces resistance against blast disease in rice lines [9]. Finally, it has to mention that the results of this study will be differed using of other isolates because of diverse effectors encoded by different isolates and their specific interactions with rice blast immunity system. Rice blast immunity is categorized in a gene-for-gene system. It explains how *Avr* genes within the pathogen connect to particular *R* genes in rice and how the absence of *R* genes will make rice susceptible [61]. Certain molecules, known as effectors, are produced by phytopathogens and encoded by virulence genes (*Avr*). These effectors are delivered directly into plant cells during the primary stage of infection and change host plant physiology. Effectors are used to promote pathogen colonization or interrupt the activation of host cells defenses. On the other hand, rice plants have consequently responded with a form of immunity that is based on the sensitivity of effectors to host resistance proteins. This immunity is called the gene-for-gene system [62].

## Conclusion

The innate immune system allows plants to react to potential pathogens in an efficient manner while reducing damage and energy costs. Photosynthesis and chlorophyll contents decrease significantly in treated susceptible varieties compared with controls. These data indicate that the pathogen is accessing the plant’s carbon sources. Thus, resistance genes should play a role in the rice immune system against blast. As no significant differences were observed in photosynthesis and its components between inoculated and non-inoculated resistant varieties, it appears that energy sources are provided to both resistant and susceptible plants but that the expression of resistance genes restricts pathogen growth and accessibility to carbon. Our findings provide evidence that the expression of the *Pikh*, *Pi9*, *Pi21* and *Osw45* genes is involved in defence responses in the leaves of rice at 31 h after the inoculation with *M*. *oryzae* pathotype P7.2. The expression level of the *Pikh* gene was constant in susceptible varieties, with symptoms that had reduced chlorophyll content and photosynthesis. Nevertheless, the *Pi9*, *Pi21* and *Osw45* genes were simultaneously up-regulated in these plants. Therefore, *Pi9*, *Pi21*, and *Osw45* are useful in the germplasm for the improvement of rice varieties against *M*. *oryzae* pathotype P7.2, whenever they are accompanied byup-regulation of the *Pikh* gene.

## Supporting Information

S1 Fig Isolation of the 330-bp-long *28SrDNA* gene from *M*. *oryzae* DNA. Lane M is the 1 kb marker, and the other lanes show the isolated *28SrDNA* gene from *M*. *oryzae*.
(TIF)

S2 Fig Standard curves obtained from 10-fold serial dilutions of the *28S rDNA* gene from blast fungus ranging from 4 to 10 log copies plotted against Cq values. E: amplification efficiency. R^2^: correlation coefficient.
(TIF)

S3 Fig Amplification plot from the real-time PCR assay of *M*. *oryzae*, obtained from the number of cycles plotted against relative fluorescence units (RFUs). (TIF)

S4 Fig Melting curves of *M*. *oryzae* for standard dilutions of the DNA template containing the *28S rDNA* gene. A melting temperature of 86.5°Cwas obtained.
(TIF)

S1 Table Simple Correlation coefficients of physiological and morphological traits in ten rice varieties. (PDF)
