# Supplementary figures and images for: Differential Gene Expression Reflects Morphological Characteristics and Physiological Processes in Rice Immunity against Blast Pathogen Magnaporthe oryzae
Source: PLoS One. 2015 May 22;10(5):e0126188. doi: 10.1371/journal.pone.0126188 (PMC4441441; doi:10.1371/journal.pone.0126188)

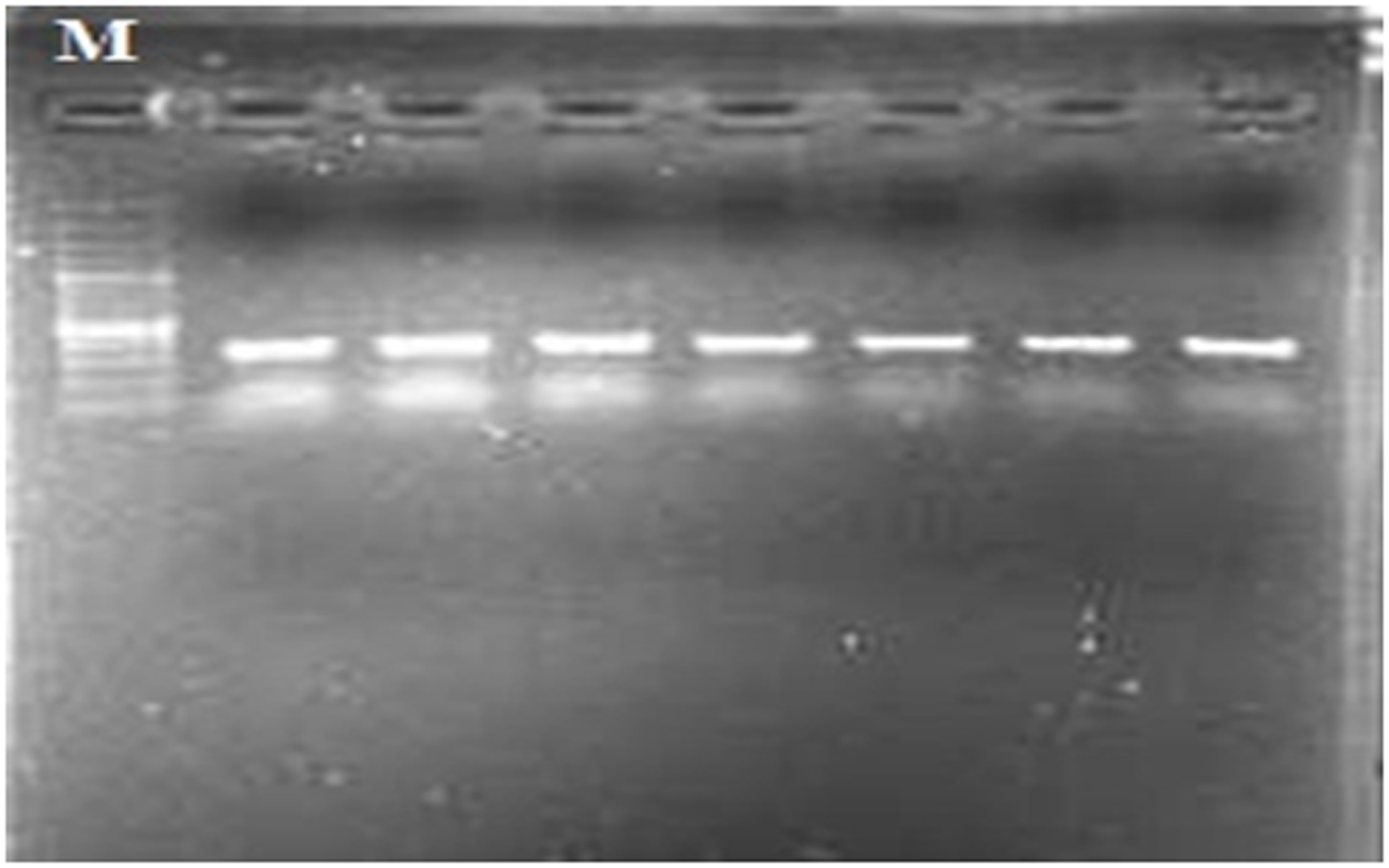

Supplement: S1 Fig — Lane M is the 1 kb marker, and the other lanes show the isolated 28SrDNA gene from M. oryzae. (TIF) [file pone.0126188.s001.tif]

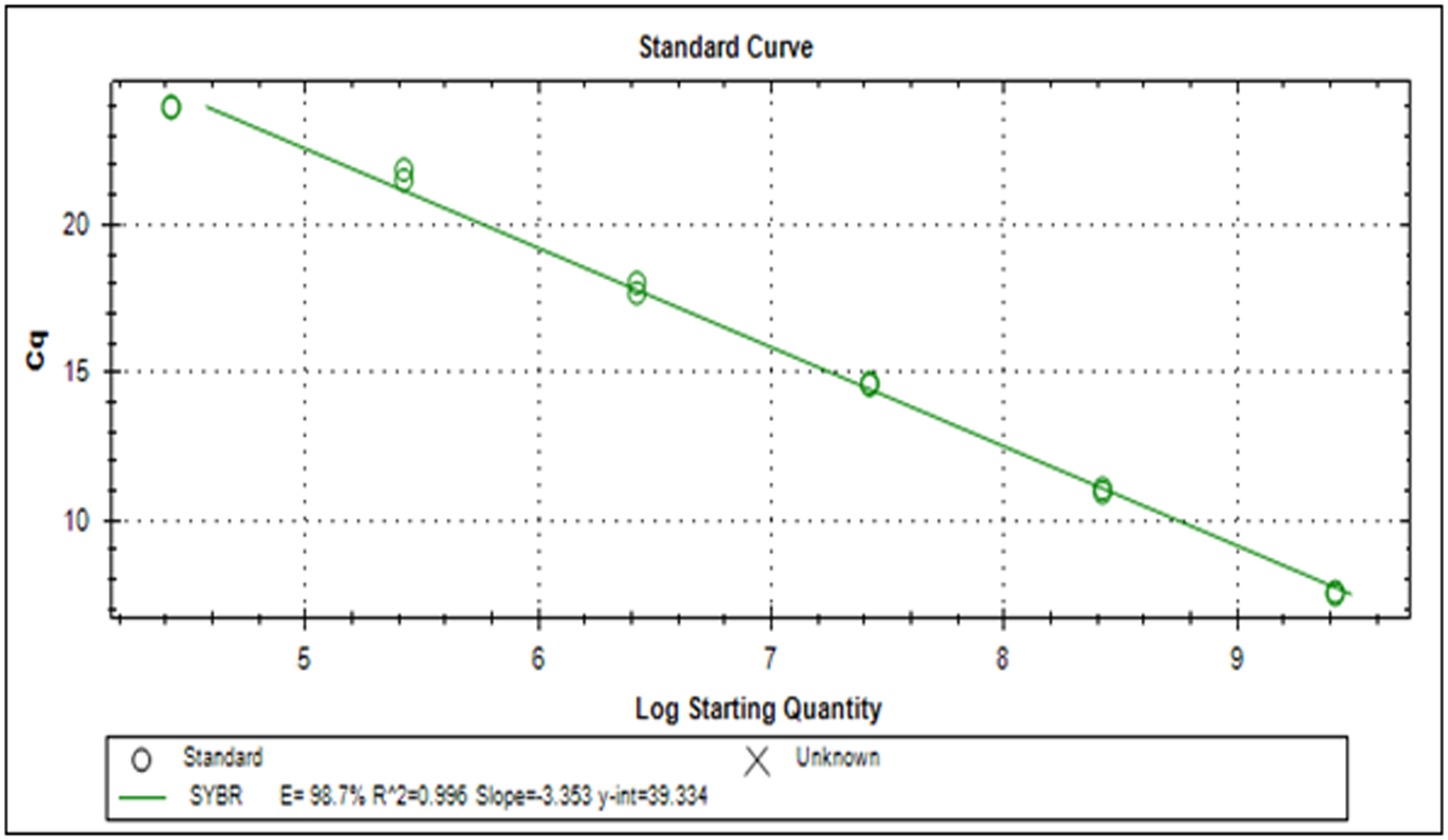

Supplement: S2 Fig — E: amplification efficiency. R2: correlation coefficient. (TIF) [file pone.0126188.s002.tif]

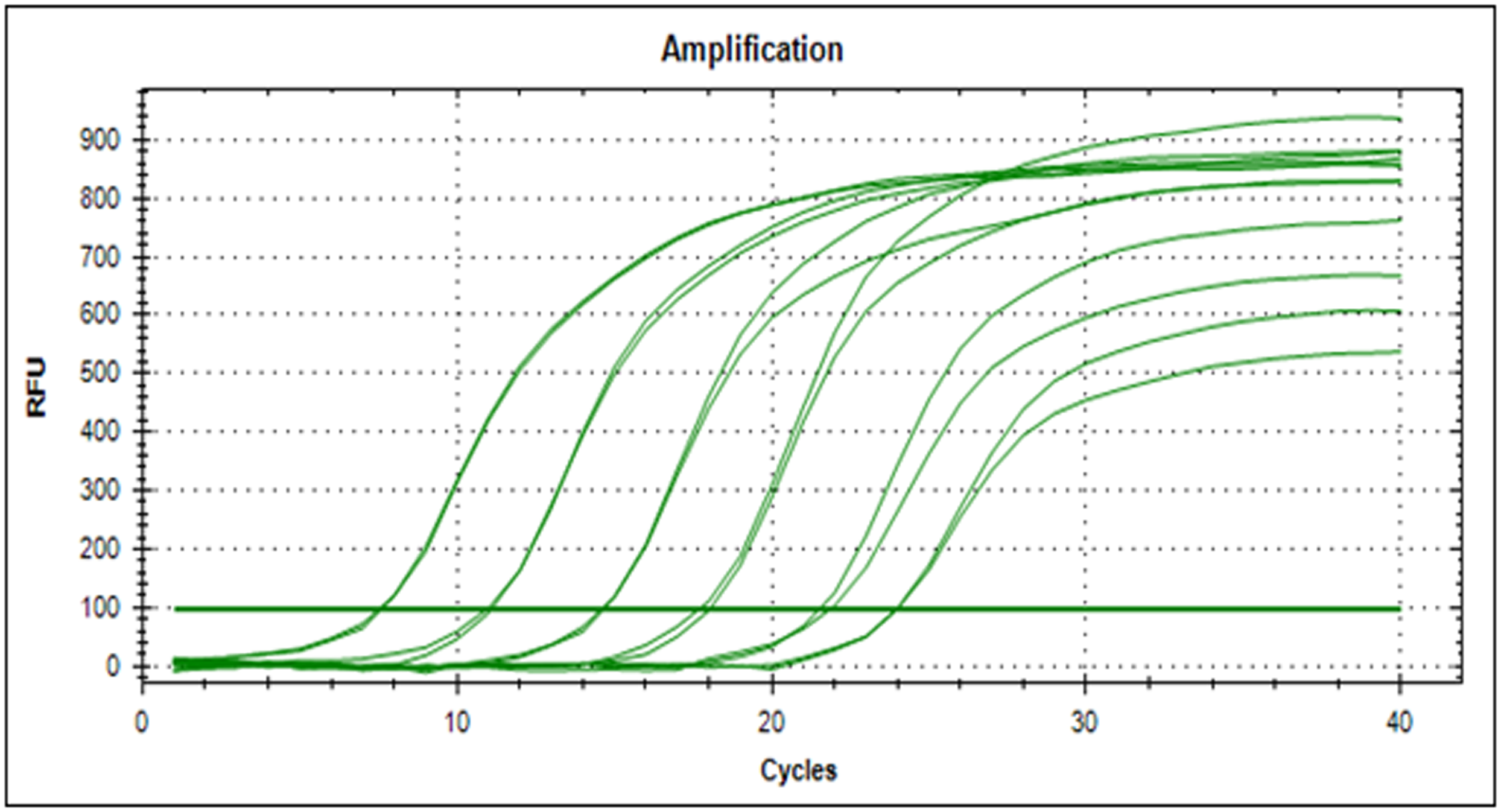

Supplement: S3 Fig — (TIF) [file pone.0126188.s003.tif]

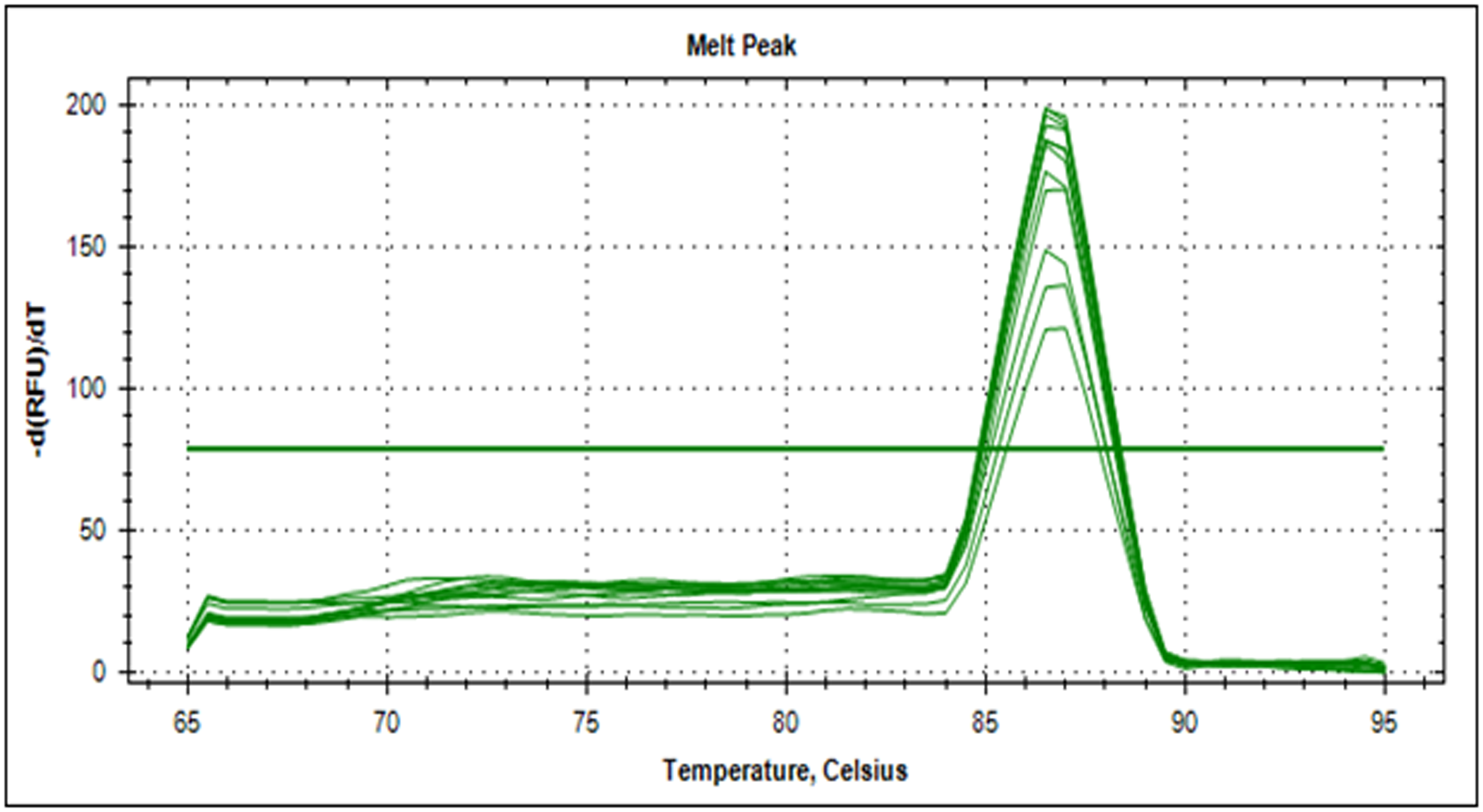

Supplement: S4 Fig — A melting temperature of 86.5°Cwas obtained. (TIF) [file pone.0126188.s004.tif]
