# Supplementary material for: Differential Gene Expression Reflects Morphological Characteristics and Physiological Processes in Rice Immunity against Blast Pathogen Magnaporthe oryzae
Source: PLoS One. 2015 May 22;10(5):e0126188. doi: 10.1371/journal.pone.0126188 (PMC4441441; doi:10.1371/journal.pone.0126188)
Supplement: S1 Table — (PDF) [file pone.0126188.s005.pdf]

S1 Table. Simple Correlation coefficients of physiological and morphological traits in ten rice varieties

	Chlo A	Chlo B	ChloT	Photo	BLT	DLA	BLD
**Chlo A**	1						
**Chlo B**	0.806**	1					
**ChloT**	0.776**	0.929**	1				
**Photo**	0.699**	0.801**	0.795**	1			
**BLT**	-0.737**	-0.852**	-0.835**	-0.916**	1		
**DLA**	-0.732**	-0.838**	-0.844**	-0.891**	0.962**	1	
**BLD**	-0.667**	-0.710**	-0.785**	-0.762**	0.789**	0.912**	1

** Correlation is significant *P < 0.01*. Chlo A: Chlorophyll A. Chlo B: Chlorophyll B. Chlo T: Total Chlorophyll. Photo: Photosynthesis. BLT: Blast lesion type. DLA: Disease leaf area. BLD: Blast lesion degree.
